# Psychological status and quality of life among primary caregivers of individuals with mental illness: a hospital based study

**DOI:** 10.1186/s12955-017-0676-y

**Published:** 2017-05-19

**Authors:** Anitha Jeyagurunathan, Vathsala Sagayadevan, Edimansyah Abdin, YunJue Zhang, Sherilyn Chang, Saleha Shafie, Restria Fauziana Abdul Rahman, Janhavi Ajit Vaingankar, Siow Ann Chong, Mythily Subramaniam

**Affiliations:** Research Division, Institute of Mental Health, Buangkok Green Medical Park, 10, Buangkok View, 539747 Singapore

**Keywords:** Depression, Anxiety, Impact, Quality of life, Primary caregivers

## Abstract

**Background:**

This study aimed to explore the psychological status and quality of life among primary caregivers of individuals suffering from various mental illnesses including early psychosis, chronic schizophrenia, depressive disorders, anxiety disorders and dementia.

**Methods:**

A total of 350 primary caregivers with relatives seeking treatment at a tertiary psychiatric hospital were recruited for this study. Socio-demographic data was obtained and the brief version of the World Health Organisation Quality of Life instrument was used to assess caregiver’s quality of life (QOL). Psychological status among primary caregivers was assessed using the General Anxiety Disorder - 7 item (GAD-7) and Patient Health Questionnaire - 9 item (PHQ-9) scales. Family Interview Schedule (FIS) was used to assess the impact of caregiving relating to social problems, interpersonal strain among family members, work related problems and financial difficulties as a result of their relative’s illness. The socio-demographic and clinical correlates of QOL, PHQ-9 and GAD-7 were examined using multiple linear and logistic regression analyses. Associations between QOL domains and psychological status was examined using multiple linear regression analyses.

**Results:**

The mean age of the primary caregivers was 49.7 years (SD = 13.2), ranging from 21 to 82 years, with a preponderance of females (67.6%), aged 50–64 years old (45.7%). Majority were of Chinese ethnicity (57.5%), had secondary level education (43.1%), were married (65.2%), and employed (64.9%). 18.3% of primary caregivers had symptoms of depression (based on PHQ-9 cut-off point of 10 or greater) while 12.7% had symptoms of anxiety (based on GAD-7 cut-off point of 10 or greater). Multiple linear and logistic regression analyses revealed that primary caregivers aged between 35-49 years and 50–64 years, unemployed, living with others, providing care to those diagnosed with dementia and who had higher FIS scores were significantly more likely to report symptoms of depression whilst those who cared for their son/daughter were less likely to be associated with symptoms of depression. Primary caregivers who had lower education, were living with others, were single or divorced/separated, were unemployed and with higher FIS scores were associated with lower QOL domain scores. Those with symptoms of depression were significantly associated with low QOL across all four domains, whilst those with symptoms of anxiety were significantly associated with low QOL in the social relationships domain.

**Conclusion:**

Psychological status of caregivers in the current study was associated with the various domains of QOL. In particular, caregivers’ symptoms of depression were significantly associated with lower QOL across all four domains of QOL whereas symptoms of anxiety were associated with lower scores in the social relationships domain. The study suggests a need to provide caregivers with social support and psycho-education to improve the QOL as well as aid in developing healthy coping strategies.

## Background

With greater awareness of human rights issues and changes both in the treatment of mental illnesses as well as in the delivery of psychiatric services, a paradigm shift has occurred with a move from hospital centred care to community-based services across most Asian countries [1]. The shift towards community care and the de-institutionalization of people with mental illness has resulted in families forming the major support system in the continual care of the mentally ill in the community [2]. Studies conducted in Asian countries showed that about 70% of people with schizophrenia live with their relatives [2] and depend on their family for care provision [3]. Despite this, family members are often inadequately prepared to be the main caregiver for their ill relatives [4] and often experience stress given that these illnesses are long-lasting and have an unpredictable course [5].

Caregivers experience psychological and emotional distress, reduction in social contact, financial difficulties, report lower life satisfaction, poor physical and mental health as a result of caregiving [6]. This chronic stress and daily hassles cause profound objective and subjective burden for caregivers of relatives with severe mental illness [7]. Research has shown psychological distress such as anxiety, depression and insomnia among caregivers of psychiatric out-patients to be twice as high as in the general population [8]. El-Tantawy et al. for instance, found that 18.33% of caregivers of schizophrenia patients as compared to 3.33% of healthy non-caregivers met the criterion for being at risk of depression [9].

Quality of life (QOL) is defined as an individual’s perception of his position in life in the context of the culture and value systems in which he lives and in relation to his goals, expectations, standards and concerns. It comprises different dimensions such as an individual’s physical, emotional, psychological and social well-being, level of independence, and environmental relationships [10]. Few research studies have reported the association between caregivers’ burden and reduction in QOL [11]. Previous studies have found that caregivers of family members with various mental illnesses experience high psychological distress, severe financial and socio-psychological problems as well as problems connected with everyday life which in turn result in a low QOL [11, 12].

Socio-demographic characteristics including age, marital status, education, employment status, and diagnosis of the ill relative have been shown to be associated with the psychological status and QOL of primary caregivers [11, 13]. Lua et al. for instance, found Malaysian caregivers of schizophrenia patients who were males, younger than 50 years old, adequately educated, employed, and receiving monthly income to report significantly better health-related QOL on the SF-36 [14]. In particular, male caregivers reported significantly higher scores in most domains except emotional well-being and general health perception, while those younger than 50 years had better QOL profiles across all domains. Those with adequate education had better scores in the domains of physical functioning, emotional well-being, energy, physical component summary (PCS) and the mental component summary (MCS), and those who were employed and receiving a monthly income had higher scores on all Short Form Survey-36 domains with the exception of role limitations due to physical health and energy or fatigue.

In contrast, Angermeyer et al., found no association between gender, diagnosis of ill relative and the QOL (assessed using the WHOQOL-BREF) of spousal caregivers of people with schizophrenia, depression or anxiety disorders [11]. Age of caregiver however, was inversely related to the physical well-being domain of QOL. With respect to psychological status, Magana et al. found younger caregiver age, and lower levels of caregivers’ education to be predictive of higher levels of caregivers’ depressive symptoms among Latino caregivers of adults with schizophrenia [13].

Singapore is a developed country in Southeast Asia with a 5.5 million multi-ethnic population comprising Chinese (74.3%), Malay (13.3%) and Indian (9.1%), while 3.3% belong to Other ethnic group [15]. The Singapore healthcare system provides services in both public and private sectors. The care of people with mental illness in Singapore rests almost entirely on the specialized mental health services in both public and private sectors [16] with an increasing emphasis on providing care within the community setting. This shift in caregiving responsibility emphasizes the importance of studying psychological status, stress, burden and QOL experienced by the caregivers. However, very few local studies have explored the psychological status and QOL of caregivers of relatives affected by mental illnesses [17]. The present study intends to examine the psychological status and QOL among caregivers of relatives suffering from mental illnesses including early psychosis, chronic schizophrenia, depressive disorders, anxiety disorders and dementia.

## Methods

### Sample description

The study used data from a cross-sectional survey that was conducted among primary caregivers of outpatients seeking treatment at the Institute of Mental Health (IMH) from July 2014 to March 2015. IMH is a tertiary care psychiatric hospital in Singapore that serves a patient population with a wide range of psychiatric illnesses.

A total of 350 primary caregivers were recruited through convenience sampling for this study. Participants in this study were Singapore residents (including Singapore Citizens and Permanent Residents) aged 21 years and above, who were primary caregivers of patients with mental illness such as early psychosis (first episode psychosis), chronic schizophrenia (those with more than 2 years of illness were considered as having chronic schizophrenia), depressive disorders, anxiety disorders, dementia and unknown mental illness (wherein the caregiver did not know the specific name of the illness). The diagnoses were reported by caregivers. The recruitment process comprised advertisements and flyers that were put up in the clinics where potential participants could easily access the details of the research project. Trained research staff was also stationed at the outpatient clinics to recruit potential participants and they would explain the study details prior to consent taking. To be included in the study, participants had to be either literate or capable of understanding English, Chinese, Malay or Tamil language, be the main caregiver and a relative of the person with mental illness, have lived with the person with mental illness for at least 6 months and be involved in providing care and support to the relative with mental health related problems. The main caregiver was defined as a person belonging to the patient’s informal support system who takes care of and is responsible for the patient, and commits most of his or her time to that task without receiving any economic retribution [18].

Ethics approval was obtained from the Institutional ethics Review Board (National Healthcare Group Domain Specific Review Board (DSRB). Written informed consent was obtained from all caregivers before their participation in the study. The questionnaire was either self-administered or administered by trained research assistant for participants who could not read and write. The questionnaire was administered either in the clinic rooms or a quiet corner in the clinic or in the research assessment rooms and it took about 45–60 min per participant. Socio-demographic questionnaire and Family Interview Schedule (FIS) were administered by the interviewer while other questionnaires were self-administered.

### Data collection

A socio-demographic questionnaire was used to obtain data such as age, gender, ethnicity, marital status, education level attained, employment status, income, living circumstances, diagnosis of ill relative, relationship of primary caregiver of ill relative, and medical history. Along with this socio-demographic questionnaire, the brief version of the World Health Organisation Quality of Life instrument (WHOQOL-BREF) was used to assess caregiver’s QOL. FIS was used to assess the impact of caregivers. Psychological status among caregivers was assessed using General Anxiety Disorder (GAD-7) and Patient Health Questionnaire (PHQ-9) scales.

The World Health Organization Quality of Life assessment-abbreviated version (WHOQOL-BREF) [19] is a 26-item, self-administered psychometrically cross-cultural instrument developed in 23 centres from developing and developed countries. This instrument has been well validated in Asian samples [20]. It is available in 19 different languages and is a subjective assessment of an individual’s perceived QOL in the past 2 weeks in four domains: physical health, psychological, social relationships and environment. It has an additional two items for assessing overall QOL and general health. All items are constructed on variations of a 5-point Likert Scale, with scores from 1 to 5, enquiring on “how much”, “how completely, “how often”, “how good” or “how satisfied” the individual felt. Domain scores are scaled in a positive direction, with higher scores denoting higher QOL except for items 3, 4 and 26 which need to be reversed scored. Since the current study focuses on the association between QOL and other variables we used the raw scores without transforming them using the approach suggested by Alshubaili et al. [21]. The Cronbach’s alpha of the different domains in the current study was as follows - Physical health 0.80, Psychological 0.82, Social relationships 0.75 and Environment 0.83. While WHOQOL-BREF has not been validated among caregivers of persons with mental illness in Singapore, it has been used among caregivers in other Asian countries such as India and Malaysia [22, 23]. The overall internal consistency of WHOQOL-BREF scale in this sample was high (Cronbach’s alpha =0.93).

Family Interview Schedule (FIS) [24] is a multi-sectional instrument that captures the perceptions of family members on several familial and cultural factors as well as relatives’ perceptions of factors that may influence the outcome of mental disorders. Four sections of the questionnaire were administered including impact, stigma, service providers, and attribution, but only the impact on informant section of the original questionnaire has been used in the current study. The section on impact of caregiving consists of four questions relating to social problems, interpersonal strain among family members, work related problems and financial difficulties as a result of their relative’s illness. Scoring is based on how caregiving has affected their life ranging from ‘almost never or not at all’ to ‘almost always or a lot’ in terms of each domain. The effect on each domain is assessed both in the past (in general) and that expected in the future. The past scores were used for analysis in the current study. The internal consistency of FIS in this sample was high (Cronbach’s Alpha = 0.74).

Patient Health Questionnaire (PHQ-9) is a 9-item self-administered instrument used to identify depression, panic disorder as well as assess stress and functional outcome of participants. The nine items assess symptoms and one question assesses functional impairment. Items are scored on a scale of 0 (not at all) to 3 (nearly every day). The PHQ-9 scale has been used in the target population i.e. caregivers of people with severe mental illness [25] and is a useful tool in assessing depression in the primary caregivers. Cut-off scores of more than 10 as reported in other studies were used to identify people with depression. A systematic review of studies using PHQ-9 concluded that it has good sensitivity and specificity for detecting depressive disorders [26]. PHQ-9 has been validated in terms of criterion validity, convergent validity and reliability, and it appears to be a valid and reliable instrument for screening depression in the primary care setting in Singapore [27]. The internal consistency of PHQ-9 in this sample was high (Cronbach’s alpha = 0.88).

Generalized Anxiety Disorder (GAD-7) is a 7-item self-administered instrument designed primarily as a screening and severity measure for generalized anxiety disorder. Each GAD-7 item is scored on a scale of 0 to 3 and has a total score range of 0 to 21. A score of 10 or greater is recommended as a cut-off point for further evaluation when screening for anxiety disorders [28]. Sensitivity and specificity of this instrument (optimal cut-off point at 10) was 89% and 82% respectively [28]. This instrument has been validated among the general population in Singapore [29]. The internal consistency of GAD-7 in this sample was high (Cronbach’s alpha = 0.89).

### Statistical analyses

All statistical analyses were performed using the SPSS version 23. Descriptive analyses were performed to calculate the frequencies and percentages for the basic socio-demographic variable and mean and standard deviation for the WHOQOL-BREF, PHQ-9, and GAD-7 variables. Multiple linear and logistic regression analyses were used to examine socio demographic correlates of QOL, PHQ-9 and GAD-7, after adjusting for all socio-demographic characteristics. Age group, ethnicity, gender, marital status, education, employment status, relationship of primary caregivers with ill relative, living arrangements, diagnoses of relative with mental health problems were included as predictors of QOL, PHQ-9 and GAD-7 scores. In order to establish psychological status and QOL attributed to burden of taking care of someone who had the mental illness, FIS scores were analysed as one of the covariates while examining the effects of socio-demographic predictors on psychological status and QOL in regression analyses. In these regression analyses, possible multi-collinearity between predictor variables was determined by obtaining the variance inflation factor (VIF). If the VIF was more than 10, multi-collinearity was considered. The model fit of logistic regression models were tested using Hosmer-Lemeshow goodness-of-fit tests. Statistical significance was evaluated at the 0.05 level using 2-sided tests.

## Results

### Study participants

The socio-demographic characteristics of the primary caregivers are presented in Table 1. Eleven participants were withdrawn from the study due to their failure to complete the questionnaires, resulting in a total of 339 participants whose data were analysed subsequently. The mean age of the caregivers was 49.7 years (SD = 13.2), ranging from 21 to 82 years, with a preponderance of females (67.6%), aged 50–64 years old (45.7%), Chinese (57.5%), secondary level education (43.1%), married (65.2%), and employed (64.9%). Majority of primary caregivers were parents (34.8%) of the ill relative, lived with their spouse (46%) and had relatives with a diagnosis of chronic schizophrenia (39.5%).The mean (SD) FIS scores was 4.28 (3.25).

**Table 1:** Socio-demographic characteristics of primary caregivers

Variable	Category	Overall	
		n	%
Age Group	21–34	63	18.6
	35–49	81	23.9
	50–64	155	45.7
	65+	40	11.8
Ethnicity	Chinese	195	57.5
	Malay	74	21.8
	Indian	65	19.2
	Others	5	1.5
Gender	Men	110	32.4
	Women	229	67.6
Marital Status	Single	87	25.7
	Married	221	65.2
	Widowed	15	4.4
	Divorced/Separated	16	4.7
Education	No formal/Primary	52	15.3
	Secondary	146	43.1
	Vocational	20	5.9
	A level/Pre-University/Diploma	67	19.8
	University and Above	54	15.9
Employment Status	Employed	220	64.9
	Students and Homemakers	92	27.1
	Unemployed	27	8
Relationship of primary caregivers with Ill relative	Spouse	80	23.6
	Parent	118	34.8
	Son/Daughter	91	26.8
	Sibling	36	10.6
	Other relatives	13	3.8
Living Arrangement	Lives alone	3	0.9
	Lives with parents	111	32.7
	Lives with Spouse	156	46
	Live with other relatives	14	4.1
	Lives with children	43	12.7
	Others	12	3.5
Diagnosis of Ill relative	Early Psychosis	19	5.6
	Chronic Schizophrenia	134	39.5
	Depressive disorders	103	30.4
	Anxiety disorder (OCD & GAD)	27	8
	Dementia	16	4.7
	Others	11	3.2
	Unknown	29	8.6
Any Medical Conditions	Yes	132	38.9
	No	207	61.1
		Mean	SD
Age of Primary Caregivers		49.7	13.18
PHQ-9 scores		4.73	5.26
GAD-7 scores		3.9	4.49
FIS scores		4.28	3.25
WHOQOL-DOMAIN1 (Physical health)		25.58	4.36
WHOQOL-DOMAIN2 (Psychological)		9.71	2.39
WHOQOL-DOMAIN3 (Social relationships)		20.44	3.83
WHOQOL-DOMAIN4 (Environment)		27.35	4.6

### Socio-demographic correlates of symptoms of depression and anxiety

18.3% of primary caregivers had symptoms of depression (based on PHQ-9 cut-off of 10 or greater) while 12.7% had symptoms of anxiety (based on GAD-7 cut-off point of 10 or greater). Socio-demographic correlates of symptoms of depression and anxiety are shown in Table 2. Multiple logistic regression analyses revealed that primary caregivers aged between 35 and 49 years and 50–64 years (compared with those 65 years and above), those who were unemployed, living with others, providing care to those diagnosed with dementia and with higher FIS scores were significantly associated with higher risk of symptoms of depression whilst those who cared for son/daughter were associated with lower risk of symptoms of depression. Those living with others and having higher FIS scores were associated with higher risk of symptoms of anxiety.

**Table 2:** Socio-demographic correlates of symptoms of depression and anxiety

Socio-demographic Variables	Patient Health Questionnaire (PHQ-9)				Generalized Anxiety Disorder (GAD-7)			
	Odd Ratio	95% CI		*p* value	Odd Ratio	95% CI		*p* value
Age Group								
21–34	7.06	0.79	63.46	.081	2.45	0.26	22.87	.430
35–49	14.53	1.96	107.86	**.009**	1.71	0.23	12.84	.604
50–64	6.37	1.06	38.44	**.043**	2.10	0.37	12.08	.404
65+	Reference							
Ethnicity								
Malay	1.75	0.67	4.53	.252	1.21	0.43	3.46	.717
Indian	0.74	0.27	2.06	.570	0.43	0.12	1.59	.207
Others	4.14	0.35	49.68	.262	1.81	0.14	23.45	.651
Chinese	Reference							
Gender								
Men	1.57	0.64	3.85	.326	1.23	0.45	3.32	.688
Women	Reference							
Marital Status								
Single	2.49	0.79	7.85	.120	1.23	0.33	4.67	.758
Widowed	0.07	0.00	1.15	.063	0.16	0.01	2.54	.193
Divorced/Separated	1.28	0.23	7.14	.779	0.36	0.04	2.98	.344
Married	Reference							
Education								
No formal/Primary	2.12	0.49	9.21	.317	2.03	0.36	11.49	.424
Secondary	1.09	0.36	3.35	.880	1.63	0.43	6.28	.474
Vocational	1.61	0.31	8.30	.571	1.03	0.13	8.00	.975
A level / Pre-university / Diploma	0.97	0.30	3.11	.955	1.29	0.32	5.18	.718
University & Above	Reference							
Employment Status								
Students and Homemakers	1.78	0.71	4.50	.220	0.75	0.26	2.20	.598
Unemployed	4.00	1.08	14.72	**.037**	2.76	0.66	11.57	.166
Employed	Reference							
Relationship of primary caregivers with Ill relative								
Spouse	0.77	0.24	2.45	.663	0.82	0.23	2.98	.763
Son/Daughter	0.08	0.02	0.40	**.002**	1.25	0.25	6.22	.785
Siblings	0.24	0.04	1.45	.120	1.98	0.31	12.83	.474
Other Relatives	0.08	0.00	1.56	.097	1.00			
Parent	Reference							
Living Arrangement								
Lives alone	4.62	0.04	509.24	.523	1.08	0.01	78.96	.971
Lives with parents	3.37	0.91	12.54	.070	0.60	0.11	3.12	.539
Lives with other relatives	0.56	0.03	10.06	.694	0.18	0.01	3.96	.276
Lives with children	2.62	0.65	10.63	.177	2.60	0.58	11.60	.212
Others	8.52	1.44	50.58	**.018**	6.95	1.15	41.81	**.034**
Lives with Spouse/partner	Reference							
Diagnosis of Ill relative								
Early Psychosis	0.85	0.18	4.06	.841	2.67	0.57	12.51	.212
Depressive disorder	1.33	0.58	3.04	.500	1.31	0.52	3.30	.570
Anxiety disorder (OCD & GAD)	1.15	0.30	4.46	.838	1.29	0.26	6.38	.754
Dementia	5.38	1.07	26.89	**.041**	1.56	0.22	10.88	.652
Others	0.12	0.01	1.37	.088	0.72	0.06	8.43	.792
Unknown	0.46	0.09	2.50	.372	0.47	0.05	4.37	.509
Chronic Schizophrenia	Reference							
FIS scores	1.41	1.26	1.59	**<.001**	1.40	1.23	1.60	**<.001**
Nagelkerke R-square	0.27				0.24			
Hosmer-Lemeshow goodness-of-fit test	274,21, *p* value = 0.8135				314.38, *p* value =0.116			

*p* value <0.05 is highlight in bold

### Socio-demographic correlates of QOL domains

The mean (SD) for the QOL domains were as follows - physical health 25.58 (4.36), psychological 9.71 (2.39), social relationships 20.44 (3.83) and environment 27.35 (4.6) respectively. Socio-demographic correlates of QOL domains are shown in Table 3. Multiple linear regression analyses revealed that primary caregivers who were widowed (β = 2.93) (compared with those were married) were significantly associated with higher scores in the physical health domain whilst those who had lower education (no formal/primary (β = −3.39), secondary (β = −1.66), vocational (β = −2.59) and A level/Pre-university/Diploma (β = −1.69) (compared to those with University or above) and had higher FIS scores (β = −-0.49) were associated with lower physical health domain scores. We also found that those living with others (β = −2.30), had no formal/primary education (β = -3.35) and with higher FIS scores (β = −0.51) were associated with lower social relationships domain scores. Primary caregivers who were single (β = −1.93) or divorced/separated (β = −1.50), those who were unemployed (β = −1.03), caring for spouse (β = −0.82), living with children (β = −1.38) and with higher FIS scores (β = −0.19) were significantly associated with lower psychological domain scores. We also found that those with no formal/primary (β = −3.74) and secondary education level (β = −2.06), those living with children (β = −2.45) and with higher FIS scores (β = −0.59) were significantly associated with lower scores in the environmental domains.

**Table 3:** Socio-demographic correlates of Quality of Life (QOL)

	Quality of Life (QOL) four domains															
	Physical health				Psychological				Social relationships				Environmental			
Socio-demographic variables	Beta co-efficient	95% CI		*p* value	Beta co-efficient	95% CI		*p* value	Beta co-efficient	95% CI		*p* value	Beta co-efficient	95% CI		*p* value
Age Group																
21–34	−0.43	−2.55	1.69	.689	0.72	−0.45	1.89	.226	−1.05	−2.87	0.76	.253	0.63	−1.50	2.76	.564
35–49	−1.02	−2.93	0.90	.297	0.69	−0.36	1.75	.197	−0.95	−2.58	0.69	.255	0.35	−1.57	2.27	.722
50–64	0.38	−1.17	1.93	.628	0.37	−0.49	1.23	.396	−0.68	−2.00	0.64	.311	0.87	−0.69	2.42	.272
65+	Reference															
Ethnicity																
Malay	−0.23	−1.41	0.95	.703	−0.28	−0.93	0.37	.398	0.42	−0.59	1.43	.415	−0.32	−1.51	0.87	.595
Indian	0.50	−0.72	1.72	.420	0.40	−0.28	1.07	.247	0.46	−0.58	1.51	.382	−0.10	−1.32	1.13	.876
Others	−0.24	−4.06	3.57	.900	1.37	−0.73	3.48	.200	1.08	−2.18	4.35	.513	−1.34	−5.17	2.50	.494
Chinese	Reference															
Gender																
Men	0.31	−0.77	1.39	.571	0.38	−0.22	0.97	.211	−0.11	−1.03	0.81	.816	−0.10	−1.18	0.99	.862
Women	Reference															
Marital Status																
Single	−0.31	−1.82	1.20	.685	−1.93	−2.76	−1.10	**<.001**	−0.79	−2.08	0.49	.227	0.28	−1.23	1.80	.713
Widowed	2.93	0.38	5.47	**.025**	−0.99	−2.39	0.42	.168	0.47	−1.71	2.65	.670	2.16	−0.40	4.72	.098
Divorced/Separated	0.16	−2.19	2.50	.895	−1.50	−2.83	−0.17	**.027**	0.94	−1.06	2.95	.357	1.08	−1.28	3.44	.368
Married	Reference															
Education																
No formal/Primary	−3.39	−5.24	−1.54	**<.001**	−0.60	−1.61	0.42	.252	−3.35	−4.93	−1.77	**<.001**	−3.74	−5.60	−1.89	**<.001**
Secondary	−1.66	−3.09	−0.22	**.024**	−0.33	−1.12	0.46	.414	−1.22	−2.44	0.01	.052	−2.06	−3.50	−0.61	**.005**
Vocational	−2.59	−4.78	−0.41	**.020**	−0.44	−1.64	0.77	.478	−0.67	−2.54	1.20	.480	−2.16	−4.36	0.04	.054
A level / Pre-university / Diploma	−1.69	−3.19	−0.18	**.028**	−0.44	−1.27	0.39	.298	−0.57	−1.86	0.72	.386	−0.95	−2.47	0.56	.217
University & Above	Reference															
Employment Status																
Students and Homemakers	−1.09	−2.24	0.07	.065	−0.16	−0.80	0.47	.614	−0.93	−1.92	0.05	.063	0.46	−0.70	1.62	.438
Unemployed	−1.11	−2.88	0.65	.216	−1.03	−2.00	−0.05	**.039**	−0.23	−1.74	1.28	.766	−0.84	−2.62	0.94	.355
Employed	Reference															
Relationship of primary caregivers with Ill relative																
Spouse	−0.33	−1.75	1.09	.646	−0.82	−1.61	−0.04	**.039**	0.05	−1.17	1.26	.940	−1.11	−2.53	0.32	.128
Son/Daughter	0.12	−1.63	1.87	.894	0.26	−0.71	1.22	.601	0.24	−1.26	1.73	.754	−0.88	−2.64	0.88	.325
Siblings	0.06	−1.99	2.11	.953	0.65	−0.48	1.78	.256	0.34	−1.41	2.10	.699	−0.34	−2.40	1.72	.747
Other Relatives	0.54	−2.20	3.29	.697	0.64	−0.87	2.15	.407	0.92	−1.43	3.26	.443	2.01	−0.75	4.77	.153
Parent	Reference															
Living Arrangement																
Lives alone	−0.39	−5.40	4.62	.880	0.42	−2.34	3.18	.763	0.28	−4.00	4.56	.898	0.91	−4.12	5.95	.721
Lives with parents	0.95	−0.72	2.62	.265	−0.09	−1.01	0.83	.844	0.70	−0.72	2.13	.334	0.25	−1.43	1.93	.770
Lives with other relatives	0.44	−2.42	3.29	.764	−0.92	−2.49	0.65	.249	−0.21	−2.65	2.22	.863	−1.36	−4.23	1.50	.350
Lives with children	−1.40	−3.17	0.36	.119	−1.38	−2.36	−0.41	**.005**	−1.45	−2.96	0.06	.060	−2.45	−4.23	−0.68	**.007**
Others	0.77	−1.84	3.39	.561	−0.80	−2.24	0.64	.276	−2.30	−4.54	−0.07	**.044**	−1.52	−4.15	1.12	.258
Lives with Spouse/partner	Reference															
Diagnosis of Ill relative																
Early Psychosis	0.62	−1.38	2.63	.541	0.47	−0.66	1.61	.412	0.59	−1.13	2.30	.501	0.81	−1.20	2.83	.427
Depressive disorder	−0.44	−1.50	0.62	.413	0.09	−0.50	0.67	.769	0.30	−0.61	1.21	.515	0.03	−1.04	1.09	.960
Anxiety disorder (OCD & GAD)	−0.67	−2.39	1.04	.439	−0.31	−1.26	0.63	.512	−1.06	−2.52	0.41	.156	−1.21	−2.93	0.51	.167
Dementia	−1.53	−3.72	0.67	.172	−0.07	−1.27	1.14	.915	−1.56	−3.43	0.32	.103	−1.51	−3.71	0.70	.179
Others	1.73	−0.99	4.45	.212	0.27	−1.23	1.77	.720	−0.53	−2.86	1.80	.654	−1.70	−4.43	1.04	.223
Unknown	0.73	−1.03	2.49	.418	−0.01	−0.98	0.96	.991	−0.32	−1.83	1.18	.675	−0.27	−2.04	1.50	.765
Chronic Schizophrenia	Reference															
FIS scores	−0.49	−0.63	−0.35	**<.001**	−0.19	−0.27	−0.11	**<.001**	−0.51	−0.63	−0.39	**<.001**	−0.59	−0.73	−0.45	**<.001**
R-squared Values (R2)	0.17				0.18				0.21				0.23			

*p* value <0.05 is highlight in bold

### QOL with psychological status

Association between QOL and psychological status variables is shown in Table 4. Multiple linear regression analyses after adjusting for socio-demographic variables, other psychological status and FIS scores revealed that those with symptoms of depression were significantly associated with low QOL across all four domains of physical health, psychological, social relationships and environment. Caregivers with symptoms of anxiety were only significantly associated with low QOL in the domain of social relationships.

**Table 4:** Association between Quality of Life domains with psychological status scales

	Quality of Life (QOL) four domains															
	Physical health				Psychological				Social relationships				Environmental			
	Beta coefficient	95% CI		*p* value	Beta coefficient	95% CI		*p* value	Beta coefficient	95% CI		*p* value	Beta coefficient	95% CI		*p* value
PHQ-9 (cut-off score > 10)	−3.27	−4.77	−1.77	**<.001**	−0.98	−1.84	−0.12	**.026**	−1.71	−3	−0.42	**.010**	−2.2	−3.73	−0.67	**.005**
GAD-7 (cut-off score > 10)	−0.84	−2.54	0.86	.334	−0.27	−1.25	0.71	.589	−1.87	−3.34	−0.41	**.013**	−1.62	−3.36	0.11	.067
Age Group																
21–34	−0.01	−2.02	1.99	.990	0.85	−0.31	2	.151	−0.73	−2.45	1	.408	0.99	−1.06	3.03	.343
35–49	−0.39	−2.21	1.43	.674	0.88	−0.17	1.93	.100	−0.58	−2.14	0.99	.471	0.8	−1.06	2.66	.397
50–64	0.77	−0.69	2.24	.300	0.49	−0.36	1.33	.261	−0.41	−1.67	0.85	.525	1.18	−0.31	2.68	.121
65+	Reference															
Ethnicity																
Malay	−0.06	−1.18	1.06	.918	−0.23	−0.87	0.41	.484	0.54	−0.42	1.51	.268	−0.18	−1.32	0.96	.755
Indian	0.4	−0.75	1.55	.496	0.36	−0.3	1.03	.282	0.33	−0.66	1.33	.511	−0.22	−1.4	0.96	.709
Others	0.38	−3.22	3.99	.835	1.56	−0.51	3.64	.140	1.58	−1.52	4.69	.317	−0.79	−4.47	2.9	.674
Chinese	Reference															
Gender																
Men	0.33	−0.68	1.35	.520	0.38	−0.2	0.97	.197	−0.11	−0.98	0.77	.812	−0.09	−1.12	0.95	.869
Women	Reference															
Marital Status																
Single	0.04	−1.38	1.47	.951	−1.82	−2.64	−1	**<.001**	−0.56	−1.78	0.67	.375	0.56	−0.9	2.02	.449
Widowed	0.2	−2.02	2.42	.857	−1.48	−2.79	−0.17	**.027**	0.86	−1.06	2.77	.379	1.03	−1.24	3.3	.372
Divorced/Separated	2.26	−0.15	4.67	.066	−1.19	−2.58	0.2	.093	−0.05	−2.13	2.03	.961	1.58	−0.88	4.05	.208
Married	Reference															
Education																
No formal/Primary	−3.01	−4.75	−1.26	**.001**	−0.48	−1.48	0.53	.354	−3.02	−4.52	−1.51	**.000**	−3.39	−5.17	−1.6	**<.001**
Secondary	−1.59	−2.95	−0.23	**.022**	−0.31	−1.09	0.47	.437	−1.11	−2.28	0.06	.062	−1.96	−3.35	−0.57	**.006**
Vocational	−2.35	−4.41	−0.28	**.026**	−0.36	−1.55	0.83	.552	−0.5	−2.27	1.28	.583	−1.96	−4.07	0.15	.069
A level / Pre-university / Diploma	−1.68	−3.1	−0.26	**.021**	−0.44	−1.26	0.38	.292	−0.54	−1.76	0.69	.390	−0.93	−2.38	0.53	.210
University & Above	Reference															
Employment Status																
Students and Homemakers	−0.91	−2	0.19	.104	−0.11	−0.74	0.52	.735	−0.86	−1.8	0.08	.073	0.56	−0.55	1.68	.323
Unemployed	−0.49	−2.17	1.19	.568	−0.84	−1.81	0.13	.089	0.25	−1.2	1.7	.733	−0.3	−2.02	1.41	.728
Employed	Reference															
Relationship of primary caregivers with Ill relative																
Spouse	−0.39	−1.73	0.95	.568	−0.84	−1.61	−0.07	**.033**	0	−1.16	1.15	.995	−1.16	−2.53	0.21	.096
Son/Daughter	−0.64	−2.33	1.05	.456	0.03	−0.94	1	.951	−0.15	−1.6	1.3	.839	−1.39	−3.11	0.34	.115
Siblings	−0.38	−2.33	1.56	.700	0.52	−0.6	1.64	.360	0.15	−1.53	1.82	.863	−0.61	−2.6	1.38	.546
Other Relatives	−0.34	−2.95	2.26	.797	0.37	−1.13	1.87	.629	0.2	−2.04	2.45	.860	1.23	−1.43	3.89	.364
Parent	Reference															
Living Arrangement																
Lives alone	0.53	−4.2	5.27	.825	0.7	−2.02	3.43	.613	1.01	−3.07	5.09	.628	1.71	−3.12	6.55	.486
Lives with parents	1.22	−0.37	2.81	.133	−0.01	−0.93	0.9	.978	0.75	−0.62	2.12	.280	0.36	−1.26	1.99	.659
Lives with other relatives	0.26	−2.43	2.96	.847	−0.98	−2.53	0.57	.216	−0.42	−2.74	1.9	.721	−1.57	−4.32	1.18	.263
Lives with children	−1.13	−2.8	0.54	.183	−1.3	−2.26	−0.34	**.008**	−1.21	−2.65	0.22	.098	−2.2	−3.9	−0.5	**.012**
Others	1.86	−0.64	4.35	.144	−0.47	−1.91	0.96	.518	−1.39	−3.54	0.76	.204	−0.53	−3.08	2.02	.683
Lives with Spouse/partner	Reference															
Diagnosis of Ill relative																
Early Psychosis	0.63	−1.26	2.53	.512	0.49	−0.64	1.61	.394	0.71	−0.92	2.35	.392	0.91	−1.03	2.85	.357
Depressive disorder	−0.28	−1.28	0.72	.580	0.14	−0.44	0.71	.645	0.42	−0.45	1.28	.344	0.16	−0.86	1.18	.761
Anxiety disorder (OCD & GAD)	−0.52	−2.13	1.1	.529	−0.27	−1.2	0.66	.571	−0.94	−2.33	0.46	.187	−1.08	−2.73	0.58	.201
Dementia	−1.01	−3.08	1.07	.340	0.09	−1.11	1.28	.884	−1.24	−3.03	0.55	.174	−1.12	−3.24	1	.298
Others	0.84	−1.74	3.43	.521	0.01	−1.48	1.49	.994	−1.13	−3.36	1.1	.318	−2.4	−5.04	0.25	.075
Unknown	0.4	−1.26	2.06	.635	−0.11	−1.06	0.85	.828	−0.58	−2.01	0.85	.427	−0.55	−2.25	1.15	.522
Chronic Schizophrenia	Reference															
FIS scores	−0.32	−0.47	−0.18	**<.001**	−0.14	−0.22	−0.06	**.001**	−0.37	−0.49	−0.25	**<.001**	−0.44	−0.59	−0.3	**<.001**
R-squared Values (R2)	0.25				0.26				0.29				0.3			

Beta coefficients were derived using multiple linear regression analyses after adjusting for socio-demographic variables, other caregiver psychological status scales and impact on informant scores

*p* value <0.05 is highlight in bold

## Discussion

The current study examined the association between psychological status and QOL among caregivers of relatives suffering from various mental illnesses. In general, 18.3% and 12.7% of primary caregivers in our study reported symptoms of depression and anxiety respectively. These rates were lower than those reported by Yıkılkan et al. who found that 58.7% of Turkish family caregivers of patients in long-term facilities met the criteria for at least mild depression (BDI score range: 8–15) and 84.1% met the criteria for at least mild anxiety (BAI score range: 8–15) [30]. In contrast, our findings mirrored those of Liang et al. who found 26.5% of caregivers of relatives with subjective cognitive decline (SCD) and cognitive impairment in Shanghai to have anxiety symptoms and 22.4% to have depressive symptoms (based on the Hospital Anxiety and Depression Scale (HADS)) [31]. It is however important to note that the rates may not be directly comparable given that the current study examined symptoms of anxiety and depressive symptoms across caregivers of relatives with various mental illness, as opposed to the aforementioned studies which looked at caregivers of specific populations (i.e., individuals with cognitive decline and patients in long-term facilities).

Previous studies which have compared depressive symptoms between caregivers and non-caregivers have found the former group to exhibit more symptoms as compared to the latter [25, 32]. For instance, Beeson et al. and Bauer et al. found primary caregivers of individuals diagnosed with probable Alzheimer’s disease or dementia to report significantly more depression [33] and anxiety than non-care givers [34]. While the current study did not explore this difference, the prevalence of depressive symptoms obtained among caregivers in this study was higher (18.3%) than that reported among primary care patients with multimorbidity (diabetes, hypertension, and dyslipidaemia) of 4.49% and 9.6% in Singapore respectively [35, 36].

With regards to socio-demographic correlates, caregivers with lower levels of education were significantly more likely to report a lower QOL in the physical, social, and environmental domains. The results were similar to other studies [13, 37] which have associated a higher level of education among caregivers with knowledge of dealing with stressful situations resulting in better QOL. Highly educated caregivers also tend to have better jobs with higher salaries which could command more resources that can be helpful in taking care of their ill relative which may also contribute to their better QOL [38]. Lower levels of education among primary caregivers is often related to lower socioeconomic status which may lead to fewer resources and handling of caregiving responsibilities on their own, which may result in poor QOL [39, 40]. Caregivers who were single or widowed had significantly poorer QOL in the psychological domain as compared with those who were married. It is possible that these caregivers faced more challenges in the absence of a spouse who could offer support and share some of the distress.

Employment was also found to have a significant association with the QOL of caregivers. Those who were employed reported a better QOL (psychological domain) than those who were unemployed. One reason for this might be that employed individuals may have a wider social network which allows them to interact with other people thereby reducing their emotional distress. Likewise, additional earnings through employment may aid in alleviating their financial distress [41]. Other studies have similarly reported higher distress among caregivers who were unemployed than those who were employed leading to a lower QOL [14, 37].

Our findings also indicated that primary caregivers’ living arrangements was significantly associated with QOL. Caregivers who lived with their children reported a lower QOL on the psychological and environment domains. Past studies have shown similar results whereby caregivers living with family members experience greater distress than those who did not live with their family members [42, 43]. Caregiver’s who live with their children may face greater stress and difficulty in coping with situations such as the ill person’s behaviour in contrast to someone who is living only with the person with mental illness. While family members may help in caregiving, it is also possible that other family members may also need care or other members may be critical of the demands placed by the person with mental illness on the relative. However, the current study did not explore these factors in detail.

Those who were caring for their spouses (ie. spousal caregivers) as opposed to parental caregivers also reported lower scores in the psychological domain. This was in contrast with a systematic review conducted by Ennis et al. who found majority of the studies to show no difference between parent and spousal caregivers of adult patients with traumatic brain injury in terms of levels of distress [44]. Serio et al. however, suggested a possible explanation to account for the observed difference between these two groups [45]. For instance, compared to parents, spousal caregivers may have lost their confidant, economic support, household co-manager and child-rearing assistant which may account for the lower scores in the psychological domain [44, 45].

In general, higher FIS scores were associated with lower scores across all four domains of QOL. In other words, caregivers who reported greater difficulties in social life, job opportunities, finances, and family relationship problems as a result of care giving had lower QoL scores. After adjusting for socio-demographic variables and FIS scores, those with depressive symptoms were significantly associated with lower QOL across all four domains of physical health, psychological, social relationships and environment while those with symptoms of anxiety were only significantly associated with low social relationships domain. This was in line with Moreno et al. who found significant correlations between caregivers’ mental health and QOL among informal caregivers of patients with dementia in Colombia [46]. In particular, caregivers with higher levels of self-reported depressive symptoms reported lower vitality and worse general health (health related QOL as measured by the SF-36).

Findings of the study should be considered in view of its limitations. The sample consisted of primary caregivers of mentally ill individuals recruited from a tertiary hospital thus limiting the generalizability to caregivers in other settings. Furthermore, this study relied on two modes of questionnaire administration (interviewer and self-administered) and the responses may be affected by a social desirability bias in the interviewer administered group. Duration of symptoms of depression and anxiety experienced among caregivers might also be important in assessing the outcome of caregiving. However this information was not collected. The information on other community agencies which support caregivers were not collected thus limiting the ability to evaluate the impact of community support on QOL among caregivers in the current study. Therefore, future studies should consider exploring this information among caregivers. In the current analysis, we did not perform *p* value adjustment because comparison between categories was only conducted against selected reference category aka binary predictor without testing further on multiple pairwise comparisons across categories which can lead to inflation of the type-1 error rate. Lastly the cross-sectional design of the study limits any causal inferences.

Notwithstanding these limitations, a significant strength of the study is the inclusion of a multi-ethnic group of primary caregivers with relatives suffering from various mental illnesses, recruited from a single site. It is also a single-phase assessment, using widely accepted questionnaires which were translated into the three languages used locally.

## Conclusion

The current study provided insight into the psychological status and QOL of caregivers looking after their relatives with various mental illnesses in Singapore. Findings elucidate the adverse outcomes that caregiving can have on the QOL and the mental health of individuals and emphasizes the need for social support and psycho-education programmes for caregivers to help them cope with distress associated with caring for a relative with mental illness. It is important that mental health professionals identify the needs of caregivers; the problems faced by them and refer them to suitable services, so as to ensure a better QOL.

## Abbreviations

QOL: Quality of Life
FIS: Family Interview Schedule
GAD-7: Generalized Anxiety Disorder
PHQ-9: Patient Health Questionnaire
VIF: Variance Inflation Factor
